# Peptide and non‐peptide mimetics as potential therapeutics targeting oral bacteria and oral biofilms

**DOI:** 10.1111/omi.12267

**Published:** 2019-08-15

**Authors:** Maryta N. Sztukowska, Mohammad Roky, Donald R. Demuth

**Affiliations:** ^1^ Department of Oral Immunology and Infectious Diseases University of Louisville School of Dentistry Louisville Kentucky

**Keywords:** antimicrobial peptide, peptidomimetics, targeted therapeutics

## Abstract

The development of the oral biofilm requires a complex series of interactions between host tissues and the colonizing bacteria as well as numerous interspecies interactions between the organisms themselves. Disruption of normal host–microbe homoeostasis in the oral cavity can lead to a dysbiotic microbial community that contributes to caries or periodontal disease. A variety of approaches have been pursued to develop novel potential therapeutics that are active against the oral biofilm and/or target specific oral bacteria. The structure and function of naturally occurring antimicrobial peptides from oral tissues and secretions as well as external sources such as frog skin secretions have been exploited to develop numerous peptide mimetics and small molecule peptidomimetics that show improved antimicrobial activity, increased stability and other desirable characteristics relative to the parent peptides. In addition, a rational and minimalist approach has been developed to design small artificial peptides with amphipathic α‐helical properties that exhibit potent antibacterial activity. Furthermore, with an increased understanding of the molecular mechanisms of beneficial and/or antagonistic interspecies interactions that contribute to the formation of the oral biofilm, new potential targets for therapeutic intervention have been identified and both peptide‐based and small molecule mimetics have been developed that target these key components. Many of these mimetics have shown promising results in in vitro and pre‐clinical testing and the initial clinical evaluation of several novel compounds has demonstrated their utility in humans.

## 1 INTRODUCTION

Periodontal diseases and dental caries are the most common biofilm‐mediated diseases of the oral cavity in both developing and developed countries. Dental caries primarily affects the hard tissues in the oral cavity and results in demineralization of tooth enamel and dentin. If left untreated, advanced lesions can induce inflammation of the pulp tissue and periapical areas of the tooth. In the U.S., 91% of adults between ages of 20 and 64 had experienced dental caries in their permanent dentition and 27% had untreated tooth decay (Dye, Thornton‐Evans, Xianfen, & Iafolla, 2015). Treatment of early stage dental caries involves restoration of the enamel using fluoride but later stage lesions require removal of the decayed hard tissue followed by restoration with a filling. Periodontitis is a chronic inflammatory disease that leads to the destruction of periodontium and ultimately results in resorption of the underlying alveolar bone and tooth loss (Darveau, 2010). The incidence of periodontal disease ranges from 30% of the population in developed countries to over 70% of the population in developing countries, with severe disease inflicting 7%–15% of human population worldwide (Eke, Dye, Wei, Thornton‐Evans, & Genco, 2012; Gera, 2000; Hugoson, Sjondin, & Norderyd, 2008). In the U.S., the total prevalence of periodontitis in adults aged 30 and older was 47.2%, representing approximately 64.7 million adults (Eke et al., 2012). Periodontal diseases are also associated with many systemic diseases and conditions, including coronary artery disease, rheumatoid arthritis, diabetes, pulmonary diseases, cancers and Alzheimer's disease (Dominy et. al., 2019; Hajishengallis, 2015; Maddi & Scannapieco, 2013; Whitmore & Lamont, 2014). Treatment of periodontal disease involves the physical removal of dental plaque from the subgingival pocket by scaling and root planning and gingival surgery to reduce pocket depth if necessary.

Although the oral cavity carries a high microbial load, incidental tissue damage or tissue damage resulting from minor surgical procedures rarely results in infection, suggesting that potent antimicrobial defence mechanisms exist in the oral cavity. An important aspect of innate immunity that may contribute to these defence mechanisms involves the secretion of antimicrobial peptides and proteins by oral epithelial cells, neutrophils, salivary secretions and gingival crevicular fluid. More than 45 types of antimicrobial peptides have been identified in saliva (Gorr, 2009). Many of these antimicrobial components exhibit a broad spectrum of activity in vitro, suggesting that they may represent promising therapeutics against oral bacteria. However, therapeutic application of antimicrobial peptides has been limited since the natural concentration of many oral antimicrobial peptides in saliva and gingival crevicular fluid is well below the minimal inhibitory concentration for many organisms and in higher doses, these peptides have shown haemolytic activity damaging mammalian cells (Aoki, Kuroda, & Ueda, 2012). In addition, antimicrobial peptides may be susceptible to proteolytic degradation and many of the organisms that are associated with periodontal disease are highly proteolytic. These limitations have spurred research to develop antimicrobial peptide mimetics in order to increase their activity and stability.

The oral cavity is colonized by a diverse group of microorganisms that can comprise over 700 species of bacteria, viruses and fungi (Dewhirst et al., 2010; Paster, Olsen, Aas, & Dewhirst, 2006). Development of this community is a dynamic process involving attachment of bacteria to oral surfaces, cohesion and communication among constituent organisms, and adaptation to the biofilm environment through direct contact, by intra‐ and interspecies signalling via soluble mediators and/or by nutrient transfer (Kolenbrander et al., 2002; Kolenbrander, Palmer, Periasamy, & Jakubovics, 2010; Kuboniwa et al., 2017). Within the oral ecosystem, bacteria are able to collectively regulate activities and functional specialization is present. With an increased understanding of the complex interspecies and host–microbe interactions that occur and contribute to the formation of the oral biofilm, new potential therapeutic targets to disrupt biofilm development have been identified. This in turn has led to the development of peptides and small molecule peptidomimetics that target specific oral bacteria. This review will summarize some of the efforts to exploit host antimicrobial peptides and the molecular processes contributing to biofilm growth to develop peptide mimetics and small molecule compounds that target oral bacteria and/or the formation of the oral biofilm.

## 2 ANTIMICROBIAL PEPTIDE‐BASED MIMETICS

### 2.1 Histatin 5 mimetics Dhvar4 and Nal‐P‐113

The histatins are a family of 12 small multifunctional proteins produced by the parotid and submandibular salivary glands (Tsai & Bobek, 1998). The histatins are generated by proteolytic cleavage of two gene products, histatin 1 and histatin 3 and function primarily as antifungal agents although they have broader antimicrobial activities as well. Histatin 5 is a cationic peptide of 24 amino acids that is generated from cleavage of histatin 3 and represents a platform that has been used to synthesize peptide mimetics with improved activity and stability. For example, dhvar4 and dhvar4a are derivatives of the C‐terminal 12 amino acids of histatin 5 (Helmerhorst et al., 1999) in which the His residues have been substituted with by hydrophobic amino acids (Phe or Leu) and the penultimate Gly replaced with Lys (Table 1). In dhvar4a, the C‐terminus was further modified by amidation. Mimetic dhvar4 (and dhvar4a) exhibits strongly enhanced antibacterial activity compared with histatin 5 against pure cultures of a broad range of oral pathogens. Viable cell counts were reduced by >3 logs for oral streptococci (*S. mutans, S. sanguis, S. salivarius*), *Actinomyces naeslundii* and *Fusobacterium nucleatum*, 2 logs for *Veillonella parvula* and 0.92 logs for *Prevotella intermedia* (Helmerhorst et al., 1999). In contrast, histatin 5 had little effect on viable cell counts (log reduction 0.0–0.3). Dhvar4 (100 µg/ml) was also tested against a seven species biofilm grown on hydroxyapatite disks in continuous culture using the organisms referenced above, as well as multispecies biofilms formed by inoculating disks with human saliva or dental plaque samples. Dhvar4 was significantly less effective in reducing viable cell counts of bacteria in the seven species biofilm (0.47–0.76 log reduction). Interestingly, dhvar4 was more effective against obligate anaerobic organisms (*F. nucleatum, V. parvula and P. intermedia*) in the plaque and saliva biofilms. For all biofilm experiments, chlorhexidine was used as a positive control and significantly reduced viable cell counts (2.5–3.0 log reductions). These results suggest that dhvar4 is more potent against planktonic cells and is significantly less active against biofilms.

**Table 1:** Antimicrobial peptide‐based mimetics

Histatin 5	DSHAKRHHGYKRKFHEKHHSHRGY
Dhvar4	KRLFKKLLFSLRKY
Dhvar4a	KRLFKKLLFSLRKY‐CONH2
P−113	AKRHHGYKRKFH‐CONH2
Nal‐P−113	Ac‐AKR‐Nal‐Nal‐GYKRKF‐Nal‐CONH2
IDR−1018	VRLIVAVRIWRR‐CONH2
HE1	RRWIRVAVILRV‐CONH2
HE2	VRLIRAVRIWRR‐CONH2
HE10	VRLI‐‐VRIWRR‐CONH2
DJK5	D‐VQWRAIRVRVIR‐CONH2
DJK6	D‐VQWRRIRVWVIR‐CONH2
SHABP	CMLPHHGACVRLIVAVRIWRR‐CONH2
MHABP	CAQAFGPNCVRLIVAVRIWRR‐CONH2
Bac8c	RIWVIWRR‐CONH2
Dermaseptin S4	ALWMTLLKKVLKAAAKAALNAVLVGANA
K_4_‐S4(1–15)a	ALWKTLLKKVLKAAA‐CONH2
C_7_‐S4(1–13)	C_7_‐WKTLLKKVLKAAA‐CONH2
C_12_‐S4(1–13)	C_12_‐WKTLLKKVLKAAA‐CONH2
L‐K6	IKKILSKIKKLLK‐CONH2
ZXR−2	FKIGGFIKKLWRSLLA
KSL	KKVVFKVKFK‐CONH2
GH12	GLLWHLLHHLLH‐CONH2
TVH19	TKRQQVVGLLWHLLHHLLH‐CONH2

By screening a library of 25 peptides that represented various portions of histatin 5, Rothstein et al. (2001) identified peptide P‐113 (Table 1), encompassing residues 4–15, as the smallest fragment of histatin 5 that retains the full anticandidal activity of the parent protein. Sajjan et al. (2001) subsequently showed that P‐113 also retained the antibacterial activity of histatin 5. Amidation of the P‐113 C‐terminus increased antimicrobial activity of the mimetic by twofold but substitution of two or more of the five basic residues in P‐113 with neutral amino acids resulted in a significant reduction in anticandidal activity, indicating that the cationic character of histatin 5 is essential for activity. Given the potent anticandidal activity of P‐113, a clinical study was undertaken using a cohort of HIV patients and showed that treatment with an oral rinse containing P‐113 was effective in reducing oral candidiasis (Helmerhorst, Oppenheim, Choi, Cheng, & Reiner, 2007). However, broad application of P‐113 therapy was limited since its activity in vitro and in vivo was significantly reduced under conditions of high osmotic strength (Helmerhorst, Hof, Veerman, Simoons‐smit, & Amerongen, 1997) and furthermore, P‐113 was susceptible to proteolytic degradation when suspended in biological fluids such as serum, saliva and sputum (Sajjan et al., 2001). One approach to increase P‐113 stability was to synthesize a derivative of P‐113 that comprised D‐amino acids (designated P‐113D) (Sajjan et al., 2001). P‐113D retained the antimicrobial activity of the parent peptide and was stable for 7 days in sputum obtained from cystic fibrosis patients, whereas the half‐life of the parent P‐113 in sputum was 2.8 min (Sajjan et al., 2001). A second approach intended to increase activity at high ionic strength involved replacing the three His residues in P‐113 with bulky hydrophobic amino acids such as Phe, β‐naphthylalanine (Nal), β‐diphenylalanine (Dip) or β‐(4,4’‐biphenyl)alanine (Bip) (Yu et al., 2011). Nal‐P‐113 and Bip‐P‐113 were shown to retain antibacterial activity at salt concentrations up to 200 mM whereas the parent P‐113 peptide lost activity at a salt concentration below 50 mM (Yu et al., 2011). Nal‐P‐113 also exhibited antimicrobial activity against oral organisms including *P. gingivalis* (MIC = 20 µg/ml), *F. nucleatum* (MIC = 40 µg/ml) and *S. gordonii* (MIC = 80 µg/ml) but consistent with the previous anti‐biofilm activity reported for the dhvar4 mimetics, Nal‐P‐113 was less potent against single or three species oral biofilms (MIC = 320–640 µg/ml and 1,280 µg/ml, respectively) (Wang, Cheng, et al., 2015). Nal‐P‐113 cytotoxicity against periodontal ligament cells and gingival epithelial cells was minimal at concentrations <320 mM but apoptosis and cell death increased significantly at higher concentrations. More recently, the activity of Nal‐P‐113 has been examined using in vivo approaches. In a rat model of periodontitis, treatment with 100 µg/ml and 400 µg/ml Nal‐P‐113 resulted in reduced *P. gingivalis*‐mediated alveolar bone loss and a reduction in *P. gingivalis* levels and in total oral microbial load (Wang, Lin, et al., 2017). In addition, a recent clinical study showed that treatment of chronic periodontitis patients with 20 µg/ml Nal‐P‐113 reduced the levels of *P. gingivalis*, *S. gordonii*, *F. nucleatum* and *T. denticola* in subgingival plaque, however, significant reductions in pocket depth, clinical attachment loss or bleeding index were not detected (Wang, Ai, et al., 2018). A possible explanation for this observation is the short duration of the study (7 days). Finally, incorporating bulky hydrophobic amino acids into P‐113 was also recently shown to enhance the LPS neutralizing activity of the peptides (Chih et al., 2019).

### 2.2 IDR‐1018, DJK and mimetics derived from bactenecin

Peptide IDR‐1018 is a 12 residue peptide derived from bovine bactenecin that was initially characterized as an innate defence regulator (IDR) peptide that functioned to modulate innate immunity, enhance the induction of chemokines and suppress harmful inflammatory responses (Rivas‐Santiago et al., 2013). The peptide exhibited only weak antimicrobial activity against *Mycobacterium tuberculosis* but significantly reduced microbial load and lung inflammation in infected animals. However, in a subsequent screen to identify peptides with anti‐biofilm activity, IDR‐1018 was shown to specifically target and kill biofilm cells more potently that other known anti‐biofilm peptides (de la Fuente‐Nunez, Reffuveille, Haney, Straus, & Hancock, 2014). IDR‐1018 was shown to interact with and degrade the stringent response mediator ppGpp and the peptide exhibited broad spectrum activity that both inhibited the formation of biofilms and eradicated mature existing biofilms formed by *Pseudomonas aeruginosa, Escherichia coli, Acinetobacter baumannii, Klebsiella pneumoniae* and *Staphylococcus aureus* at concentrations that had no effect on planktonic cells. A series of peptide analogues highlighted several functionally important residues of IDR‐1018 (de la Fuente‐Nunez et al., 2014). Interestingly, reversing the sequence of IDR‐1018 in peptide HE1 (Table 1) had little effect on anti‐biofilm activity. In contrast, altering the hydrophobic LIVAV sequence between residues 3 and 7 of the peptide by substituting R for V^5^ (peptide HE2, Table 1) or deleting VA^5,6^ (peptide HE10, Table 1) reduced activity against Gram‐negative organisms but increased anti‐biofilm activity against Gram‐positive organisms for HE10. de la Fuente‐Nunez et al. (2015) subsequently synthesized and tested a panel of D‐enantiomer peptides based on the known properties of IDR‐1018. Two D‐peptides, DJK5 and DJK6 (Table 1) exhibited improved broad spectrum anti‐biofilm activity relative to IDR‐1018, likely as a result of being resistant to proteolytic degradation.

The anti‐biofilm activity of IDR‐1018 against oral biofilms has also been examined. Wang, Fuente‐Nunez, Shen, Haapasalo, and Hancock, (2015) inoculated hydroxyapatite and saliva‐coated hydroxyapatite discs with samples of supragingival dental plaque. Planktonic cultures in broth media were also grown. Treatment of planktonic cultures with up to 80 µg/ml IDR‐1018 did not inhibit growth. In contrast, the addition of 1, 5, or 10 µg/ml IDR‐1018 to the plaque suspension at the time of inoculation resulted in a dose and time‐dependent reductions of biofilm biovolume and biofilm cell death of up to 10‐fold after 3 days. Little to no difference was observed with biofilms grown on hydroxyapatite or saliva‐coated discs. In addition, significant reductions in biofilm biovolume and an increase in cell death occurred after treatment of pre‐formed 3 day biofilms with IDR‐1018 for as little as 1 min. A parallel approach was used to evaluate the activity of DJK5 peptide against oral biofilms derived from supragingival plaque. Similar results were obtained in that DJK5 was significantly more potent against biofilm cultures than planktonic cells. For these experiments, IDR‐1018 was used as a positive control and in virtually all cases, DJK5 exhibited increased potency relative to IDR‐1018 (Zhang, Wang, Hancock, Fuente‐Nunez, & Haapasalo, 2016). The activity of IDR‐1018 was also examined against planktonic and pre‐formed biofilm cultures of *C. albicans* and a *Candida* clinical isolate (CI) (Freitas et al., 2017). The MIC for planktonic *C. albicans* and CI cultures was 32 µg/ml and 64 µg/ml, respectively. In contrast, the minimum biofilm inhibitory concentration (MBIC) was 4 µg/ml and 8 µg/ml, respectively. To assess the potential therapeutic utility of IDR‐1018, the activity of the peptide was assessed using a mouse candidemia model. Animals were infected with 10^6^ *C. albicans* cells and after 24 hr, mice were treated daily with IDR‐1018 at 10 mg/kg body weight. Treated mice showed a significant increase in survival, with 50% of the animals surviving after 8 days. These animals also had reduced candidal burden in the kidneys relative to control mice. In contrast, all of the control animals treated with buffered saline died after 6 days (Freitas et al., 2017).

Recently, IDR‐1018 has been further modified in order to specifically target the peptide to enamel (Yang et al., 2019). To accomplish this, IR‐1018 was synthesized containing either a high affinity or a medium affinity hydroxyapatite‐binding heptapeptide (Gungormus et al., 2008) at its N‐terminus to generate peptides SHABP and MHABP respectively (see Table 1). Consistent with the prior activity profile of IDR‐1018, neither SHABP nor MHABP inhibited the growth of planktonic supragingival cultures. Both peptides significantly reduced biofilm biovolume and increased killing of biofilm cells upon both short‐ (3–9 min) and long‐term (1–3 days) exposure (Yang et al., 2019), however, SHABP was more effective than MHABP or the control IDR‐2018 peptides. Finally, to determine if anti‐biofilm activity occurred with biofilms growing on other material surfaces that may be present in the oral cavity, the activity of DJK5 and IDR‐1018 peptides against biofilms grown on hydroxyapatite and titanium surfaces was compared. In both cases, DJK5 was more effective and killed more bacteria than IDR‐1018, however, there was no significant difference in the extent of killing by either peptide on hydroxyapatite versus titanium surface (Wang, Haapasalo, Gao, Ma, & Shen, 2018). In summary, these results suggest that IDR‐1018 and some of its derivative peptide mimetics, especially DJK5 and DJK6, may represent novel therapeutics for treating chronic biofilm infections at a variety of tissue sites and in the oral cavity.

Peptide Bac8c is also a mimetic that was derived from bactenecin (Table 1) and is broadly active against a range of oral organisms including mutans streptococci, various commensal streptococcal species, *Actinomyces* sp. and Lactobacilli (Ding et al., 2014). MICs ranged from 16–32 µg/ml for oral streptococci and 8–16 µg/ml for *Actinomyces* species, approximately 16‐fold lower than observed with the parent bactenecin peptide. Lactobacilli were more resistant to Bac8c and displayed MICs of 64–128 µg/ml, although *L. fermenti* was as susceptible as the *Actinomyces.* The killing kinetics of Bac8c against *S. mutans* was both time‐ and dose‐dependent; incubation with 2 × MIC, 4 × MIC and 8 × MIC resulted in no viable cells after 240, 60 and 15 min, respectively. Bac8c displayed minimal toxicity against human gingival fibroblasts at concentrations <128 µg/ml with short exposure times (<60 min) but affected cell proliferation and membrane integrity at higher concentrations or upon prolonged exposures.

### 2.3 Dermaseptin K_4_‑S4 (1‑15)a

Dermaseptin S4 is a member of a large family of antimicrobial peptides ranging in size from 28 to 34 amino acids that are expressed in amphibian skin (Navon‐Venezia, Feder, Gaidukov, Carmeli, & Mor, 2002). The peptide displays broad spectrum activity against a range of bacteria, fungi and parasites but is also highly toxic towards human erythrocytes. To maintain the antimicrobial properties of the peptide and limit its toxicity, several truncated peptide mimetics have been synthesized and evaluated (Feder, Dagan, & Mor, 2000). Dermaseptin K4‐S4(1‐15)a (see Table 1) has been tested against planktonic cultures of a variety of oral organisms (Altman et al., 2006), including several strains of *S. mutans, S. sobrinus*, Lactobacilli, *A. viscosus, A. actinomycetemcomitans, F. nucleatum* and *P. gingivalis*. The MICs for these organisms ranged from 5 to 20 µg/ml and K4‐S4(1‐15)a was significantly more active against these organisms than the human antimicrobial peptide LL37 (Altman et al., 2006). However, *P. gingivalis* was resistant to K4‐S4(1‐15)a. Although K4‐S4(1‐15)a was susceptible to gingipain‐mediated cleavage, a D‐enantiomer analogue that was resistant to cleavage retained activity against *S. mutans* and other organisms at the level of the L‐peptide but was still inactive against *P. gingivalis* (Bachrach et al., 2008). K4‐S4(1‐15)a was also active against surface‐attached *S. mutans* and inhibited the formation of *S. mutans* biofilms, albeit with lower effectiveness (MBIC = 50 µg/ml) than against planktonic cultures. K4‐S4(1‐15)a exhibited even lower potency against a mature *S. mutans* biofilm, which required 500 µg/ml to eradicate (Altman et al., 2006).

To further improve antimicrobial activity and reduce potential toxicity, several peptide analogues were synthesized in which N‐terminal amino acids of K4‐S4(1‐15)a were replaced with short fatty acids (Porat, Marynka, Tam, Steinberg, & Mor, 2006). Deletion of 1–3 N‐terminal amino acids resulted in a progressive loss of antibacterial activity and a reduction in α‐helical character of the peptide, however, replacing the deleted residues with fatty acids resulted in recovery of antimicrobial activity, faster bacteriocidal kinetics and restoration of α‐helical structure. The most potent analogues possessed heptanoic (C_7_‐S4(1‐13) or aminododecanoic acid (NC_12_‐S4(1‐13) moieties replacing the N‐terminal Ala‐Leu residues of K4‐S4(1‐15)a (see Table 1). These analogues exhibited MICs between 2 and 9 µM for *B. cereus, S. aureus, P. aeruginosa* and *E. coli* and between 8 and 32 µM for oral organisms *S. mutans, A. viscosus* and *A. actinomycetemcomitans*. In addition, treatment of the polymicrobial flora present in human saliva with 100 µM C_7_‐S4(1‐13) resulted in a decrease in CFUs of >3 logs within 2 min (Porat et al., 2006). Finally, mature single species *S. mutans* or *A. viscosus* biofilms were eradicated upon 18 hr exposure to 100 µM C_7_‐S4(1‐13). These results suggest the combined N‐terminal truncation and acylation may functionally improve K4‐S4(1‐15)a and related cationic antimicrobial peptides and their derivatives.

### 2.4 L‐K6 peptide

Temporin‐1CEb is a naturally occurring 12‐residue peptide that was isolated from skin secretions *Rana chensinensis*. The peptide exhibited bactericidal activity against Gram‐positive organisms but was weakly active against Gram‐negative bacteria and like many natural antimicrobial peptides, temporin‐1CEb exhibited significant haemolytic activity. To counter these limitations, Shang, Li, et al. (2012) and Shang, Sun, Wang, Wei, and Sun (2012) designed several temporin‐1CEb analogues with increased cationic character and decreased hydrophobicity by substituting Lys residues for neutral and/or non‐polar amino acids of the peptide. The most potent analogue, L‐K6 (Table 1), was active against both Gram‐positive and Gram‐negative organisms and exhibited minimal haemolytic activity. More recently, the activity of L‐K6 against oral organisms was examined (Shang et al., 2014). L‐K6 possessed both bactericidal and fungicidal activity against *S. mutans, S. salivarius, S. sanguinis, L. acidophilus, F. nucleatum* and *C. albicans* with MICs ranging from 3.1 to 6.26 µM and MBCs ranging from 12 to 25 µM. To assess the killing kinetics against *S. mutans* and *C. albicans*, cultures were exposed to 0.5–8 × MIC L‐K6 for various times. No viable cells remained after 90 min exposure to 4 × MIC, or after 60 min exposure to 8 × MIC L‐K6. In addition, L‐K6 potently inhibited the formation of *S. mutans* biofilms (MBIC – 3.1 µM) and was bactericidal against an established *S. mutans* biofilm (MBBC = 6.26 µM). L‐K6 also exhibited anti‐inflammatory activity and significantly reduced IL‐8 and TNF‐α levels in LPS‐stimulated THP‐1 cells. L‐K6 toxicity towards THP‐1 cells was minimal at <5 µM but moderately reduced viability and proliferation of cells at 10–60 µM.

### 2.5 ZXR‐2 peptide

Peptide ZXR‐1 encompasses the N‐terminal 16 residues of mauriporin, a 48 amino acid peptide isolated from the venom gland of the Moroccan scorpion *Androctonus mauritanicus* and was initially synthesized as an anti‐cancer peptide that induced apoptosis in a variety of cancer cell lines (Zhou et al., 2016). ZXR‐1 forms an amphipathic α‐helix with clearly defined hydrophilic and hydrophobic surfaces, with the exception that Lys^14^ resides on the hydrophobic face of the peptide. In an attempt to improve its activity, Zhou et al. (2016) synthesized a new peptide, ZXR‐2, in which Lys^14^ was replaced with Leu (Table 1). Interestingly, while this single change increased overall cytotoxic activity, it also altered the mechanism of action of the peptide. ZXR‐1 functioned as a pro‐apoptotic peptide whereas ZXR‐2 disrupted the cell membrane leading to lysis. As a result, Chen et al. ( 2017) evaluated ZXR‐2 as an antibacterial peptide against a panel of 11 Lactobacillus species, four oral streptococcal species and *P. gingivalis*. *L. fermentum* and *L. mucosae* were the most susceptible organisms (MIC = 2 and 8 µM, respectively), *L. casei* was moderately susceptible (MIC = 16 µM) and the remaining Lactobacilli was resistant to ZXR‐2 activity (MIC ≥ 32 µM). *S. mutans, S. sobrinus* and *S. gordonii* exhibited MICs in the range of 8–16 µM and the MICs for *S. sanguis* and *P. gingivalis* ranged from 16 to 32 µM. No viable cells remained after 5 min exposure of *S. mutans* or *S. sobrinus* to 4 x MIC ZXR‐2 while viability of *P. gingivalis* was lost after exposure for only 2 min. Two approaches were utilized to test the effectiveness of ZXR‐2 against *S. mutans* biofilms, the first in which ZXR‐2 was added to the inoculum and the second where the peptide was added to a mature 1 day biofilm. ZXR‐2 potently inhibited the formation of *S. mutans* biofilms (MBIC = ~9 µM) but an established mature biofilm was resistant to the peptide (MBIC ≥ 128 µM). Confocal analysis of treated biofilms showed that only cells on the surface of the mature biofilm were affected by ZXR‐2 and that the overall biofilm architecture was not altered by peptide treatment. This suggests that ZXR‐2 poorly penetrates the *S. mutans* biofilm and thus may be relatively ineffective for eliminating or reducing dental plaque.

### 2.6 Designer synthetic α‐helical peptides KSL and GH12

Naturally occurring antimicrobial peptides often have several limitations for use as potential therapeutic agents in that they are often easily degraded, the peptides in their active form can exhibit significant levels of toxicity towards host tissues and the size of many naturally occurring peptides makes them expensive to manufacture at scale. To overcome these limitations, de novo synthetic antimicrobial peptides have been designed and developed. For example, Zelezetsky and Tossi (2006) described a sequence template approach to design potent artificial α‐helical peptides and optimize their activity and similar approaches have been applied in the design of peptides that target oral organisms.

Hong et al., 1998 used a combinatorial chemistry approach to design and develop a series of novel antimicrobial decapeptides, the most potent of which was designated KSL (Table 1). The KSL decapeptide represents the minimal length that is necessary for the interaction of an amphipathic α‐helical peptide with membrane‐associated phosphatidylcholine liposomes (McLean, Hagaman, Owen, & Krstenansky, 1991). KSL was shown to irreversibly inhibit the growth of *C. albicans* with a MIC of 0.78 µg/ml and also showed potent antimicrobial activity against broad range of Gram‐positive and Gram‐negative bacteria (e.g., *S. aureus, S. epidermitis, M. luteus, E. coli* and *P. aeruginosa*) with MICs ranging from 0.78 µg/ml to 6.25 µg/ml. Further studies showed that KSL exhibited minimal toxicity, lacks haemolytic activity of many naturally occurring peptides and did not induce cell death or compromise the membrane integrity of human gingival fibroblasts (Concannon et al., 2003). More recently, Liu, Wang, et al. (2011b) examined the activity of KSL against a variety of oral bacteria. In general, KSL was less effective against these organisms than the species examined by Hong et al. (1998). *S. mutans* and *L. acidophilus* were the most susceptible oral organisms, exhibiting MICs of 62.5 µg/ml, 10–90‐fold higher than the MICs reported by Hong et al. (1998). The other oral bacteria that were examined, including *S. gordonii, P. gingivalis, P. intermedia, F. nucleatum, C. albicans* and *A. actinomycetemcomitans* were more resistant to KSL, with MICs ranging from 125 to 1,000 µg/ml. Despite its moderate activity against *S. mutans*, treatment of planktonic cells with 2 × MIC KSL resulted in a 5‐log decrease in viable cells after exposure for 30 min. KSL also inhibited *S. mutans* biofilm formation, with an MBIC of 62.5–125 µg/ml but it was less effective against a mature 1‐day‐old *S. mutans* biofilm, which required 250–500 µg/ml peptide to reduce viability by 50%. Thus, although KSL represents one of the first de novo synthetic peptides, it possesses relatively low activity against cariogenic and other oral bacteria.

Wang, Fan, et al., 2017 suggested that KSL was ineffective against oral bacteria because it may not adopt the optimal structure of a cationic α‐helical peptide and based on the de novo template described by Zelezetsky and Tossi (2006), designed and synthesized three peptides, GH8, GH12 and GH16 with the sequence of GLLW‐(HLLH)_1‐3_. Structural characterization of the peptides indicated that GH8 did not assume strong α‐helical structure in solution whereas GH12 and GH16 exhibited high helical content. Each peptide was subsequently tested for activity against a panel of oral organisms including *S. mutans, S. gordonii, S. sanguinis, L. acidophilus, L. casei, A. vicosus, A. naeslundii*. GH8 exhibited poor activity for all of the organisms (MICs ranged from 32 to >512 µg/ml) and GH16 was more effective against Lactobacilli (MIC = 8 µg/ml) but was poorly active against the other organisms (MICs from 32 to >512 µg/ml). In contrast, GH12 (Table 1) exhibited potent antimicrobial activity against all of the organisms, with MICs = 4–8 µg/ml and MBCs = 8–32 µg/ml. Treatment of planktonic *S. mutans* or *L. acidophilus* with 4 × MBC GH12 resulted in a 6 log reduction in viable cells after 20 min. Even at a sub‐inhibitory dose of 0.5 × MIC, GH12 inhibited acid production, EPS synthesis and biofilm formation of *S. mutans* and significantly down‐regulated the expression of virulence genes including *ldh, gtfBCD, vicR, liaR* and *comDE* (Wang, Wang, et al., 2018). Subsequently, Jiang et al. (2018) showed that treatment of a three species biofilm comprising *S. mutans, S. gordonii* and *S. sanguinis* with 8 µg/ml GH12 reduced the abundance of *S. mutans* and reduced the level of water‐insoluble glucan (Jiang et al., 2018). Interestingly, as the abundance of *S. mutans* decreased in the treated biofilms, the level of *S. gordonii* increased and became the dominant organism in the biofilm by 48 hr.

Recently, GH12 was fused with peptide TD7 (TKREEVD) derived from the hydrophilic C‐terminal region of amelogenin which functions to promote hydroxyapatite nucleation and the growth of enamel crystals (Li et al., 2014) in order to create a bifunctional peptide with both antibacterial and mineral promoting activity (Wang et al., 2019). The initial fusion protein exhibited poor antibacterial activity but substituting Gln for Glu^4,5^ and Val for Asp^7^ in TD7 to generate peptide TVH19 (Table 1) significantly increased activity against *S. mutans*. The TVH19 fusion peptide was stable in saliva for 12 hr and was non‐toxic towards human oral keratinocytes. To examine the ability of TVH19 to promote remineralization, enamel slices derived from bovine incisors were first incubated in an acidic buffer and then transferred to remineralization buffer containing 1 × MBC TVH19 or 1,000 ppm NaF. The TVH19‐treated and NaF‐treated groups exhibited significantly higher surface hardness than the deionized water‐treated control group. In addition, the radiopaque remineralizing zone of the enamel slice was thicker on samples treated with TVH19 or NaF but did not change significantly on the water‐treated control slices. Significantly greater mineral deposition and shallower lesions were observed in the TVH19‐treated group or NaF‐treated group than in the water‐treated control (Wang et al., 2019). These results suggest that bifunctional fusion peptides may both target *S. mutans* and promote remineralization of initial caries and have utility in the treatment of dental caries.

## 3 TARGETED PEPTIDE MIMETICS

Although the oral microbiome may contain >700 bacterial species, a relatively small number of species have been associated with the common oral biofilm‐mediated diseases, caries and periodontitis. In addition, some of the organisms that are associated with periodontal disease have been suggested to induce dysbiosis in the gut microbiome (Olsen & Yamazaki, 2019). The remaining oral organisms are benign commensals although some species may play a beneficial role in the host (Walker et al., 2018). Caries and periodontal disease are generally treated using procedures that reduce both pathogens and commensals. In some cases, periodontitis can be treated using antibiotics, most of which are also broad spectrum indiscriminate antimicrobial agents. The non‐specific nature of these treatment regimens has several potential undesirable side effects, including increased chance for secondary opportunistic infections and/or the development of drug resistance. This has stimulated research to develop novel pathogen‐targeted, highly specific antimicrobials that function to reduce or eliminate organisms that are associated with disease without significantly affecting commensal populations.

### 3.1 STAMPs

Some early attempts to create targeted therapeutics involved the use of monoclonal antibodies as a targeting moiety, or generated fusion proteins where an antimicrobial peptide was linked to a bacterial recognition domain (Qiu et al., 2003; Qiu, Zhang, Wang, & Wu, 2005). Eckert et al. (2006) developed a new class of specifically (or selectively) targeted antimicrobial peptides (STAMPs) comprised of a small species‐targeting peptide linked to a broad spectrum antimicrobial peptide domain. One of the first functional STAMPs, designated C16G2 (Table 2A), utilized a 16‐mer portion of the *S. mutans* competence‐stimulating peptide (CSP) as the targeting domain fused to a flexible triglycine linker and antimicrobial peptide G2, a 16‐residue fragment of the antimicrobial peptide novispirin G10. C16G2 exhibited potent antibacterial activity against several *S. mutans* strains (MICs = 3–5 µM) but was significantly less effective against *S. sanguinis* and *S. gordonii* (MICs = 19 and 24 µM, respectively). Upon short exposure (1 min), C16G2 was >20‐fold more active against planktonic *S. mutans* than the G2 peptide alone. In contrast, there was no difference in C16G2 or G2 killing for either of the commensal streptococci, confirming the selectivity of C16G2. Similar results were found after exposing single species biofilms for 1 min to C16G2 or controls (CSP or G2 peptides); biofilm growth of the commensal species was indistinguishable from an untreated biofilm, whereas biofilm growth of *S. mutans* was significantly inhibited after exposure to C16G2 but not the control peptides. Furthermore, short‐term treatment with C16G2 but not control peptides reduced *S. mutans* levels in a mixed species community in saliva (Eckert et al., 2006) and recolonization of C16G2‐treated saliva biofilms by *S. mutans* was inhibited (Li et al., 2010). These results indicate that short‐term exposure to C16G2 is effective in reducing *S. mutans* populations in both pure culture and a mixed species community. Finally, C16G2 was shown to selectively kill *S. mutans* in a human saliva‐derived multispecies community (Guo et al., 2015). Interestingly, re‐growth of the treated microbial community showed that species with metabolic dependency with *S. mutans* (e.g., *Veillonella atypica*) were drastically reduced in abundance whereas commensal streptococcal species and other species associated with health became dominant (Guo et al., 2015). This result suggested that C16G2‐mediated removal of *S. mutans* may redirect the structure of the oral microbiome towards a commensal predominate community.

**Table 2:** Targeted peptides and mimetics

A								
C16G2	TFFRLFNRSFTQALGKGGGKNLRIIRKGIHIIKKY							
SAPP	NIFKKNVGFKK							
B								
BAR peptide	LEAAPKKVQDLLKKANITVKGAFQLFS							
combinatorial N^1182^	LEAAPKKVQDLLKKA**ZIXX**KGAFQLFS							
combinatorial T^1184^	LEAAPKKVQDLLKKA**XIZX**KGAFQLFS							
combinatorial V^1185^	LEAAPKKVQDLLKKA**XIXZ**KGAFQLFS							
CR‐BAR	LEAAPKKVQD**C**LKKANITVKGAFQ**C**FS 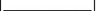							
CR2‐BAR	**C**EAAPKKVQDLLKKANITVKGAFQ**C**FS 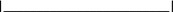							
C								
Promoted adherence		N	I	T	V	K		
		R			I			
		K			F			
		H			W			
		S						
		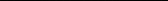						
Reduced adherence		D		D	D			
		P		P	P			
		G		G				

C16G2 is relatively stable in phosphate‐buffered saline (PBS) for over 20 hr at 4°C and for at least 4 hr at 25°C, and exhibited a half‐life of approximately 19 min in human saliva (Sullivan et al., 2011), suggesting that C16G2 could be formulated in a basic PBS mouth rinse to evaluate its activity in vivo in humans. In a pilot clinical study, patients swished with a 10% sucrose solution and subsequently with either PBS or with a PBS containing 0.04% C16G2 daily for 4 days. Patients using the placebo rinse showed a daily increase in *S. mutans* levels relative to baseline during the 4‐day period whereas the *S. mutans* levels for the group using the 0.04% C16G2 rinse showed no increase in *S. mutans* despite the daily sucrose challenges. At the end of the study, *S. mutans* levels decreased by almost 1 log compared to baseline for those treated with C16G2 but a 6.7 log increase in *S. mutans* occurred in the placebo group. In addition, the resting plaque pH was significantly higher and lactic acid production was significantly lower after C16G2 treatment. Subsequently, a Phase 2 clinical trial was conducted in 2017–2018 to evaluate the safety, tolerability and effectiveness of varnish and dental strip formulations of C16G2 (ClinicalTrials.gov Identifier: NCT03196219). Results from this study are expected to be reported by November 2020.

The basic synthetic scheme used to generate C16G2 was also applied to optimize each of the domains contributing to activity (He et al., 2010) and to generate other novel STAMP analogues (He, Anderson, Shi, & Eckert, 2009). He et al. (2010) used a combinatorial approach to evaluate a series of targeting peptides, linkers and killing peptides to improve the potency and killing kinetics of STAMPs that target *S. mutans* (He et al., 2010). This approach generated a diverse number of STAMP sequences and identified mechanisms that may limit STAMP effectiveness as well as strategies to overcome these limitations. In addition, by modifying the linker domain, multiple targeting sequences that are specific for different organisms were synthesized (He et al., 2009). This opens the possibility of using a single STAMP to simultaneously eliminate several species from multispecies cultures or biofilms. Finally, these general approaches are not only limited to the development of potential therapeutics that target *S. mutans*, but can be generally applied to any organisms for which a suitable targeting moiety has been identified.

### 3.2 Streptococcal‐derived Anti‐*P. gingivalis* peptide (SAPP)

The interaction of *P. gingivalis* with *S. cristatus* has been reported to reduce the expression of *fimA* and reduce *P. gingivalis* biofilm formation without affecting its planktonic growth (Christopher, Arndt, Cugini, & Davey, 2010; Xie, Lin, Wang, Wu, & Lamont, 2007). This interaction has been subsequently shown to be mediated by a protein–protein interaction between the *S. cristatus* protein ArcA encoding arginine deiminase and *P. gingivalis* RagB and the product of the PGN_08056 gene (Ho, Lamont, & Xie, 2017a). Interestingly, repression of *fimA* expression does not require enzymatic activity. These results highlight the potential of ArcA to target *P. gingivalis* and attenuate virulence. By using a peptide array generated from the ArcA sequence, Ho et al. (2017a) identified five ArcA‐derived peptides that directly interact with *P. gingivalis*, the most potent of which was NIFKKNVGFKK representing residues 249 – 259 of ArcA. A synthetic peptide comprising this sequence, designated SAPP (Table 2A), inhibited the expression of *fimA* as well as several other *P. gingivalis* virulence genes including *mfa1*, *kgp*, *rgpA*/*B* and *rgpA* with IC_50_ values ranging from 15 to 20 µM. Incubation of SAPP (24 µM) with *P. gingivalis* reduced the formation of monospecies biofilm on a saliva‐coated surface by up to 70% after 48 hr (Ho, Lamont, & Xie, 2017b). In addition, the formation of dual species biofilms between *P. gingivalis* and *S. gordonii* was reduced in the presence of SAPP and furthermore, treatment of a pre‐existing in vitro dual species biofilm resulted in a significant reduction in adhered *P. gingivalis*. This phenotype is consistent with the repression of *mfa* and *fimA* since both of these fimbrial proteins are involved in the interaction of *P. gingivalis* with *S. gordonii*. A similar reduction in *P. gingivalis* abundance was observed when a multi‐species plaque‐derived biofilm was incubated with SAPP (Ho et al., 2018). Interestingly, the abundance of other potential periodontal pathogens, including *T. forsythia, F. nucleatum* and *T. denticola* were also reduced by SAPP, however, the levels of *A. actinomycetemcomitans* and streptococci were unchanged. Finally, SAPP affected other fimbrial‐ and gingipain‐mediated phenotypes of *P. gingivalis* including inhibiting the invasion of epithelial cells, reducing cell surface gingipain activity, and inhibiting the ability of *P. gingivalis* to selectively impair the production of IL‐8 (Ho et al., 2017b). SAPP is stable in PBS for >24 hr but was gradually degraded over 48 hr in saliva. The peptide also exhibits minimal toxicity towards human oral keratinocytes or periodontal ligament cells (Ho et al., 2018). These characteristics suggest that SAPP may be compatible for formulations for oral applications in humans.

### 3.3 SspB Adherence Region—BAR peptide mimetics

Polymicrobial synergy between *P. gingivalis* and commensal streptococci is well established (Hajishengallis & Lamont, 2016; Lamont & Hajishengallis, 2015) and *P. gingivalis* and commensal streptococci such as *S. gordonii* are often isolated together from supra‐ and subgingival plaque (Eren, Borisy, Huse, & Mark Welch, 2014; Griffen et al., 2012; Slots & Gibbons, 1978). Furthermore, a streptococcal transcriptomic signature has been associated with periodontal disease (Yost, Duran‐Pinedo, Teles, Krishnan, & Frias‐Lopez, 2015). Previous work suggested that the initial colonization of the oral cavity by *P. gingivalis* may involve adherence of *P. gingivalis* to commensal streptococci and that adherence is mediated by a protein–protein interaction between the minor fimbrial antigen (Mfa) of *P. gingivalis* and antigen I/II (AgI/II) of streptococci (Chung, Demuth, & Lamont, 2000; Lamont et al., 2002; Park et al., 2005). Structure function studies also identified a discrete motif in AgI/II that was essential for *P. gingivalis* adherence to streptococci. Comparison of this region to the corresponding sequence in AgI/II from *S. mutans*, which does not adhere to *P. gingivalis* identified key residues (i.e., NITVK) that defined the selectivity of adherence (Demuth, Irvine, Costerson, Cook, & Lamont, 2001). Daep, James, Lamont, and Demuth, 2006) subsequently showed that a synthetic peptide, designated BAR (see Table 2B), comprising the AgI/II motif, functioned as a potent competitive inhibitor of *P. gingivalis*/streptococcal adherence, prevented the formation of dual species biofilms in vitro (IC_50_ = 1.3 µM) (Daep, Lamont, & Demuth, 2008) and significantly reduced *P. gingivalis* virulence in a mouse model of periodontitis (Daep, Novak, Lamont, & Demuth, 2011). To further define the structural constraints of NITVK that are important for interaction with *P. gingivalis*, a combinatorial approach was undertaken and three peptide libraries were synthesized in which one of the functionally active residues of NITVK (N^1182^, T^1184^, or V^1185^) was replaced by each of the 19 common amino acids (cysteine excluded). The remaining two amino acid positions were randomized by using equimolar mixtures of the 19 common amino acids during synthesis. Thus, each combinatorial library contained 19 distinct peptide mixtures that differed only in the amino acid residue that occupied the “z” position (see Table 2B) and each of the 19 mixtures in a given library contained 361 different peptide sequences, arising from randomization of the two amino acids occupying the “x” positions. As shown in Table 2C, substitutions of positively charged residues for N^1182^ and hydrophobic residues for V^1185^ improved adherence. Substitutions for T^1184^ showed little increase in *P. gingivalis* adherence (Daep et al., 2006). These observations were subsequently confirmed with a synthetic peptide containing N/R^1182^ and V/I^1185^ substitutions which exhibited 2.5‐fold greater inhibitory activity (IC_50_ = 0.5 µM) than BAR (Daep et al., 2008).

Substitution of Pro or Gly for N^1182^ or V^1185^ reduced *P. gingivalis* adherence (Table 2C), suggesting that the α‐helical character of NITVK was important for activity. Since short peptides are highly flexible in solution, Daep et al. constructed a conformationally constrained peptide to limit structural flexibility around the NITVK motif by introducing a disulfide bond (CR‐BAR, Table 2B). Unexpectedly, this peptide was 13‐fold less effective than the parent BAR peptide (Daep at al., 2008). It was subsequently determined that introducing Cys for L^1177^ disrupted a second motif (VQDLL) that was essential for *P. gingivalis* adherence (Daep et al., 2008). Indeed, the VQDLL motif, flanked by Lys residues, resembled the core LXXLL consensus sequence of the nuclear receptor (NR) box, a eukaryotic protein–protein interaction domain. Thus, BAR may represent the prokaryotic counterpart of the eukaryotic NR box (Daep et al., 2008). A second constrained peptide (CR2‐BAR, Table 2B), in which both VXXLL and NITVK remain intact, exhibited > 2‐fold increased activity relative to BAR, confirming the role of the VXXLL motif and suggesting that secondary structure of BAR is important for activity.

Potential obstacles for developing potential peptide therapeutics for oral applications include the high cost of synthesis and their susceptibility to proteolytic degradation. This is especially a concern for peptides that target periodontal organisms, many of which are highly proteolytic. Indeed, BAR contains five Lys residues and is likely to be a substrate for the Lys‐gingipain. One approach to address this potential limitation involved the synthesis of an analogue of BAR in which D‐Lys residues replaced the five L‐Lys in the peptide. This peptide was stable when incubated with *P. gingivalis* or with purified Kgp and exhibited a twofold increased specific inhibitory activity (IC_50_ = 0.7 µM) relative to the parent peptide (Daep, Novak, Lamont, & Demuth, 2010). However, although this peptide exhibited increased stability and activity, it was expensive to synthesize. These limitations for BAR and other peptide active agents have led several groups to explore the development of small molecule peptidomimetics as stable and cost‐effective alternatives.

## 4 SMALL MOLECULE INHIBITORS TARGETING ORAL BACTERIA

### 4.1 Meta‐phenylene ethylene (mPE)

Meta‐phenylene ethylene (mPE) is a novel non‐peptide mimetic whose design was based on the amphiphilic structure of magainin, an antimicrobial peptide that was initially isolated from the skin of the African frog *Xenopus laevis* (Zasloff, 1987). mPE exhibits antimicrobial activity against several oral pathogens, including *P. gingivalis*, *S. mutans* and *A. actinomycetemcomitans,* exhibiting MICs within the range of 0.4 µg/ml for *A. actinomycetemcomitans* to 2.5 µg/ml for *P. gingivalis* (Beckloff et al., 2007). Incubation of planktonic *S. mutans* with 10 x MIC mPE (~5 µg/ml) resulted in a 2 log reduction of viable organisms after 0.5 min exposure and no viable cells remained after 1 hr. In addition, the growth of in vitro* S. mutans* biofilms formed in the presence of sucrose was significantly inhibited by 2.5 µg/ml mPE and the incubation of established *S. mutans* biofilms with 50 µg/ml mPE resulted in a 3 log reduction of viable biofilm cells. mPE remains active in the presence of human saliva and its activity also extends to fungal pathogens, as mPE was shown to inhibit the growth of several *Candida* species in the concentration range of 0.5 to 1 µg/ml.

More recently, the activity of mPE was tested using a biofilm model against two bacteria associated with periodontitis, *A. actinomycetemcomitans* (Kaplan, Meyerhofer, & Fine, 2003) and *P. gingivalis* (Davey, 2006). Metabolic activity and microbial biomass measurements demonstrated that mPE rapidly killed *A. actinomycetemcomitans*, reducing overall metabolic activity by 60% after 2 hr exposure to mPE (Hua, Scott, & Diamond, 2010). Similarly, a decrease in both metabolic activity and viable biofilm bacteria was observed upon exposure of *P. gingivalis* biofilms to 4–8 µg/ml of mPE (Hua et al., 2010). Interestingly, 2 µg/ml of mPE also inhibited interleukin 1β‐induced secretion of IL‐8 in gingival epithelial cells in a dose‐dependent manner and functioned as an anti‐inflammatory agent in infected tissues, similar to the functions of the L‐K6 mimetic. The antibacterial activity of mPE against both planktonic and biofilm cultures along with its ability to suppress cytokine production in gingival tissue and lack of cytotoxicity suggests that mPE may represent a novel broad spectrum potential therapeutic against a variety of oral pathogens.

### 4.2 Peptidomimetics of BAR peptide

An alternative approach to generate stable inhibitors of *P. gingivalis*/streptococcal adherence in a cost‐effective manner involved the design and synthesis of small molecule mimetics of BAR peptide. Patil, Tan, Demuth, and Luzzio (2016) selected the 2,4,5‐trisubstituted oxazole scaffold as the peptidomimetic model of the NITVK motif in BAR peptide. The oxazole ring comprised a central torus where two aromatic rings, located at the 4 and 5 positions of the heterocycle, bear hydrophobic residues in the form of halogens, alkoxy groups or alkyl groups to mimic the essential hydrophobic character of this functional motif (Daep et al., 2006). The NITVK mimetics also contained an azide moiety that facilitated coupling to the VXXLL mimetic compounds. The initial VXXLL mimetics comprised a simple aromatic ring to mimic the hydrophobic character of the VXXLL amphipathic helix. These compounds also contained an alkynyl group to facilitate coupling to the NITVK azide group via the click reaction (Takayama, Kusamori, & Nishikawa, 2019) to produce the first generation 1,2,3‐triazole‐based inhibitors of *P. gingivalis* adherence. A total of 50 first generation compounds were tested for the inhibition of *P. gingivalis* adherence and formation of dual species biofilms and four compounds exhibited potent activity (IC_50_ = 5–8 µM) (Patil et al., 2016). These compounds were subsequently shown to inhibit adherence of *P. gingivalis* to streptococci in the presence of *F. nucleatum*, a bridging organism that can independently interact with both *P. gingivalis* and oral streptococci. In addition, the most potent first generation compound was capable of disrupting a pre‐existing three species biofilm (Tan, Patil, Luzzio, & Demuth, 2018). All four of the active first generation mimetics were subsequently shown to inhibit *P. gingivalis* virulence in vivo and exhibited minimal levels of toxicity towards various human and mouse cell lines (Tan et al., 2018). Recently, Patil, Tan, Demuth, and Luzzio (2019) reported the synthesis of second generation 1,2,3‐triazole‐based inhibitors which utilized a 1,3,5‐trisubstituted‐2,4,6‐triazine scaffold to mimic the VXXLL motif. This scaffold was based on a structure that was used to mimic the core LXXLL motif in the eukaryotic NR box. The most potent second generation peptidomimetics showed improved activity relative to first generation compounds (IC_50_ = 2–4 µM), most likely because the 1,3,5‐trisubstituted‐2,4,6‐triazine scaffold more closely mimics the VXXLL motif than the aromatic ring used in first generation compounds. These second generation mimetics exhibited minimal toxicity towards human gingival keratinocytes (Patil et al., 2019) and significantly reduced *P. gingivalis*‐mediated alveolar bone loss in infected mice (D. R. Demuth, unpublished). Thus, stable small molecule mimetics of BAR peptide have been developed and represent cost‐effective potential novel therapeutics that may limit *P. gingivalis* colonization of the oral cavity.

### 4.3 Structure‐based and functional screening of small molecule libraries

The functional screening of small molecule libraries and/or in silico structure‐based small molecule screens are common approaches to identify novel active agents in modern drug discovery. Liu, Worthington, Melander, and Wu (2011a) screened a library of 506 compounds based on nitrogen‐rich marine alkaloids to identify agents that inhibit *S. mutans* biofilm formation. Eight compounds were identified that inhibited biofilm formation by *S. mutans* at concentrations of ≤4 µM but had little effect on monospecies biofilms of *S. sanguinis* or *S. gordonii*. All of the active compounds shared structural characteristics and contained either a 2‐aminoimidazole or 2‐aminobenzamidazole scaffold. The most potent compound, 2A4, exhibited a monospecies MBIC_50_ of 0.94 µM and also inhibited planktonic growth of *S. mutans* (IC_50_ = 2 µM). In multispecies biofilms, 5 µM 2A4 reduced the abundance of *S. mutans* in the biofilm by 89% but reduced the level of commensal organism by only 20%. Treatment with 2A4 was shown to reduce the expression of three biofilm‐associated genes in *S. mutans* and reduced cell surface expression of AgI/II and GTF but had no effect on their analogues in commensal streptococci. Subsequently, Pan, Fan, Wu, Melander, and Liu (2015) demonstrated that treatment with a 2‐aminoimidazole/triazole conjugate prevented *S. mutans* biofilm accumulation in a mouse biofilm model whereas significant *S. mutans* accumulation occurred in control animals.

The same small molecule library was screened by Wright, Wu, Melander, Melander, and Lamont (2014) to identify inhibitors of *P. gingivalis/S. gordonii* biofilm formation. Interestingly, two of the 2‐aminoimidazole or 2‐aminobenzamidazole compounds identified by Liu, Worthington, et al. (2011a) also appeared to inhibit the formation of *P. gingivalis/S. gordonii* biofilms, the most potent of which was 2A4 (IC_50_ = 3.4 µM). The inhibitors identified had no effect on planktonic growth of *P. gingivalis* but did repress the expression of *fimA* and *mfa* encoding the major and minor fimbrial subunit proteins. Thus, 2‐aminoimidazoles or 2‐aminobenzamidazoles may represent broad spectrum anti‐biofilm compounds that function in part by altering the expression of genes that are essential for biofilm formation.

A structure‐based screen has also identified small molecules that target *S. mutans* GtfC. Ren et al. (2015) screened approximately 150,000 compounds against the crystal structure of the glucosyltransferase domain of the *S. mutans* GtfC protein and identified 2‐(4‐methoxyphenyl)‐*N*‐(3‐{(2‐(4‐methoxyphenyl)ethyl)imino}‐1,4‐dihydro‐2‐quinoxalinylidene)‐ethanamine as a potential Gtf inhibitor. To confirm its activity, in vitro approaches showed that the compound inhibited EPS synthesis and reduced the abundance of viable *S. mutans* cells in a biofilm by 79% at 10 µg/ml. In addition, treatment of *S. mutans*‐infected rats with the compound significantly reduced the incidence and severity of smooth and sulcal‐surface caries in vivo and reduced *S. mutans* biomass in dental plaque. A similar structure‐based screen of a library of 500,000 small molecules was conducted by Zhang et al. (2017) and identified seven inhibitors of GtfC, the most potent of which (G43) inhibited *S. mutans* biofilms by 85% at 12.5 µM. G43 significantly reduced glucan production and inhibited both GtfB and GtfC by 80%. The compound had no effect on planktonic growth or viability of *S. mutans*, commensal streptococci, *A. actinomycetemcomitans* or *A. viscosus* and did not affect biofilm formation of *S. sanguinis* or *S. gordonii*. In contrast, G43 significantly reduced the abundance of *S. mutans* in mixed species biofilms. Finally, treatment with G43 significantly reduced caries scores in a rat model of dental caries and did not exhibit overt toxicity in the treated animals. Thus, both functional and structure‐based small molecule screens identified targeted, highly potent compounds that represent potential novel anti‐*S. mutans* therapeutics.

The screens described above identified anti‐biofilm compounds that had little or no effect on planktonic cell growth, however, small molecule libraries can also be useful for identifying bactericidal compounds that target essential gene products. For example, Xu et al. (2011) developed a system to predict essential genes in *S. sanguinis* and subsequently applied this system to *P. gingivalis* W83, resulting in the identification of 212 essential genes. One of these genes encoded *meso*‐diaminopimelate dehydrogenase (*m*‐Ddh) that functions in the lysine biosynthetic pathway of *P. gingivalis* and is essential for protein and cell wall biosynthesis. Experimental inactivation of the gene encoding this enzyme (PG0806) was subsequently shown to be lethal (Stone et al., 2015). Thus, m‐Ddh represents an ideal target for therapeutic intervention. Stone et al. (2015) used a structure‐based screen to identify potential inhibitors of m‐Ddh by screening the ZINC 3D database (Irwin & Schoichet, 2005) and initially identified 11 commercially available potential inhibitors. Of the 11 compounds, four resulted in >90% inhibition of m‐Ddh when incubated with purified enzyme at a concentration of 3 mM and exhibited IC_50_ values for the inhibition of enzymatic activity between 157 µM and 1.1 mM. In the three most active compounds (4, 5 and 6), each contained a sulfonamide scaffold and were subsequently tested for inhibition of *P. gingivalis* planktonic growth. MIC/MBC for compounds 4, 5 and 6 were 250/374 µM, 167/254 µM and 2821/ND µM respectively. At 5 × MIC, compound 4 reduced *P. gingivalis* CFU by 2 logs after incubation for 6 hr, whereas no viable cells remained (5 log reduction) after 2 hr incubation with compound 5. Thus, while these compounds specifically targeted *m*‐Ddh, they possess only moderate bactericidal activity, suggesting that analogous structures and/or optimization of the sulfonamide scaffold might be required to improve whole cell inhibition.

## 5 FUTURE DIRECTIONS AND CONCLUDING REMARKS

A variety of approaches have been pursued to develop novel potential therapeutics that are active against and/or target oral bacteria. The structure and function of naturally occurring antimicrobial peptides from various sources have been exploited to develop numerous peptide mimetics that show increased activity, stability and other desirable characteristics relative to the parent peptides. In addition, as our understanding of the molecular mechanisms that contribute to the formation of the dental biofilm has increased, new potential targets for therapeutic intervention have been identified and both peptide and small molecule mimetics have been developed that target these key components. Many of these mimetics have demonstrated potent activity in pre‐clinical laboratory testing and in various animal models of oral disease, however, for the most part their activity against the more complex oral microbial communities that occur in vivo have not been thoroughly investigated. That said, the current hypothesis that oral disease arises from the disruption of normal host–microbe homoeostasis suggests that targeting specific organisms that initiate or promote the formation of dysbiotic communities may represent a viable and effective therapeutic strategy. In addition, peptide mimetics or small molecule agents may be useful administered after a professional prophylaxis to direct the re‐establishment of a microbial community that is associated with health. Future studies to further develop these active agents will likely begin to focus on developing suitable formulations for oral delivery, additional toxicity testing and evaluating stability and pharmacokinetics in the oral environment. The C16G2 STAMP has already been formulated in an oral gel, in a dental varnish and in oral strips. The initial clinical evaluation of these formulations indicated that a single varnish application outperformed multiple gel applications and resulted in a significant reduction in *S. mutans* populations. Further Phase II clinical testing is currently ongoing. It is likely that additional peptide and/or small molecule mimetics will undergo clinical evaluation in the future.
